# Nanoparticles Fabricated on Carbon Nanofibers Through Dual-Solvent-Assisted Microwave Carbothermal Shock

**DOI:** 10.3390/ma19204402

**Published:** 2026-10-20

**Authors:** Quynh Le, Fnu Joshua, Alex Burnstine-Townley, Yuen Yee Li Sip, Lei Zhai

**Affiliations:** 1 NanoScience Technology Center, University of Central Florida, Orlando, FL 32826, USA; quynh.le@ucf.edu (Q.L.); fnu.joshua@ucf.edu (F.J.); al694059@ucf.edu (A.B.-T.); yuenyee.lisip@ucf.edu (Y.Y.L.S.); 2 Department of Chemistry, University of Central Florida, Orlando, FL 32826, USA

**Keywords:** microwave carbothermal shock, carbon nanofibers, dual-solvent synthesis, antisolvent precipitation, copper nanoparticles, nickel nanoparticles, carbon nanotubes, rapid thermal processing

## Abstract

Metal nanoparticles supported on carbon nanofibers (CNFs) are promising for catalytic and electrochemical applications, but controlling their size, dispersion, and phase during rapid thermal processing remains challenging. Here, we report a dual-solvent microwave carbothermal shock (CTS) method for producing Cu- and Ni-containing nanostructures on CNFs under ambient-pressure conditions. Ethanolic metal nitrate precursors were deposited on pristine CNFs, and hexane was introduced as an antisolvent before microwave irradiation. At 0.05 M Cu(NO_3_)_2_, hexane addition decreased the mean Cu-containing particle diameter from 50 ± 20 nm to 33 ± 12 nm and improved dispersion. Varying precursor concentration further altered particle morphology and aggregation. For Ni, single-solvent processing yielded aggregated nanoparticles averaging 43 ± 16 nm, whereas dual-solvent processing produced a dense carbon nanotube network with an apparent mean filament diameter of 20 ± 7 nm. Microscopy and elemental mapping supported the association of Ni-containing particles with the nanotubes. X-ray diffraction identified crystalline face-centered cubic Cu and Ni, while X-ray photoelectron spectroscopy indicated oxidized surface species. Hexane may additionally supply carbon during Ni-assisted nanotube growth, although its contribution requires confirmation. This approach provides a rapid, tunable route to supported metal nanoparticles and hierarchical carbon nanostructures without continuous external inert or hydrocarbon gas flow.

## 1. Introduction

Metal nanoparticles (MNPs) supported on carbon nanofibers (CNFs) are attractive catalytic materials for energy conversion, environmental remediation, and chemical synthesis because they combine nanoscale metal sites with conductive, high-surface-area, and thermally stable carbon supports [1,2,3]. CNFs can facilitate the generation of well-dispersed nanoparticles during fabrication and electron transport during catalytic reactions [3,4]. MNPs can be deposited onto carbon-based supports in wet and dry processes. The wet processes, including hydrothermal and solvothermal synthesis, wet impregnation and recution, and electrodeposition, involve aqueous solutions [3,4,5,6]. Hydrothermal and solvothermal methods can provide effective control over nucleation and crystal growth, but they commonly require elevated temperatures, pressurized reaction vessels, extended processing times, and subsequent drying or thermal treatment [3,5]. Wet impregnation and reduction are comparatively simple and versatile; however, solvent evaporation and post-treatment can promote particle aggregation and broaden the particle-size distribution [3]. Electrodeposition enables precise control over metal loading and coating thickness on conductive substrates, but it is limited by the need for electrically conductive supports and careful control of electrochemical conditions [4].

Dry-process approaches can deposit MNPs onto carbon-based supports without the use of bulk liquid solvents, offering advantages such as reduced solvent handling, cleaner interfaces, and compatibility with continuous processing. Common methods include physical vapor deposition, chemical vapor deposition, atomic layer deposition, sputtering, and thermal evaporation, in which metal atoms or volatile precursors are transported through the gas phase and deposited directly onto the carbon surface [6,7,8]. These techniques can provide good control over film thickness, surface coverage, and, in some cases, nanoparticle size and composition. However, they typically require vacuum or controlled-atmosphere equipment, can be relatively costly, and may have limited throughput or difficulty achieving uniform deposition throughout thick or porous carbon substrates. In addition, excessive deposition can lead to continuous films rather than discrete nanoparticles, while high-temperature processing may alter the structure or surface chemistry of the carbon supports [6,7,8]. Achieving rapid synthesis while maintaining precise control over particle size, dispersion, and morphology remains a persistent challenge for both wet and dry processes.

Carbothermal shock (CTS) methods, including microwave and Joule heating approaches, use short thermal exposures to promote precursor conversion and nanoparticle formation while limiting prolonged coarsening [9,10,11,12,13]. In Joule heating, precursors are distributed on conductive carbon supports, and rapid heating drives decomposition, reduction, and nucleation. Reported implementations reach temperatures of approximately 1800–2000 K, with heating and cooling rates on the order of 10^5^ K s^−1^ in suitably controlled systems [11,13]. Rapid cooling can kinetically restrict sintering and phase separation, enabling small, composition-controlled or metastable nanoparticles. The resulting particle morphology and phases depend on precursor loading, support chemistry, temperature, exposure duration, and heating and cooling rates [9,10,11,12,13].

Microwave heating provides non-contact energy input to carbon-based supports and can produce rapid localized heating without conventional bulk furnace heating [14,15,16,17]. The resulting thermal history depends on microwave coupling, support properties, and heat dissipation, making precursor migration and particle coarsening important processing considerations [9,14,16]. Defect engineering has been used to improve microwave absorption and nanoparticle formation [9]. Atmosphere control is also important because metal and metal-oxide formation depend on the precursor and reaction environment [15,16]. It is noted that microwave heating has been used in wet processes where ligand capping and polymer stabilization are additional approaches to particle-size control in solution-based nanoparticle synthesis, including microwave-assisted polyol methods [10,17,18]; these should be distinguished from CTS on a carbon scaffold.

Previous work demonstrated the formation of silver nanoparticles on carbon-fiber-based supports using an ethanol–hexane precursor system followed by microwave CTS [19]. Building on that work, the present study extends the dual-solvent microwave CTS strategy to Cu and Ni precursors under ambient-pressure conditions. The formation of small, well-dispersed particles requires control of precursor distribution and growth while limiting extensive coalescence and oxidation during conversion. In the present approach, ethanolic metal nitrate precursors are deposited on CNFs, and hexane is introduced as an antisolvent. The proposed role of hexane is to reduce precursor solubility and favor more finely dispersed precursor-rich domains on accessible CNF surfaces. Subsequent microwave irradiation promotes precursor conversion and nanoparticle formation over a short processing interval, without continuous external inert or hydrocarbon gas flow. Cu(NO_3_)_2_ and Ni(NO_3_)_2_ were selected to examine how metal identity, precursor concentration, and hexane volume affect morphology, dispersion, and phase evolution. The Cu system produced supported Cu-containing nanoparticles, whereas the Ni system promoted carbon nanotube (CNT) formation. The resulting structures were characterized by SEM, TEM/HRTEM, STEM-EDS, XRD, and XPS.

## 2. Materials and Methods

Materials. Polyacrylonitrile (PAN homopolymer; average molecular weight, 150,000), N,N-dimethylformamide (DMF, Certified ACS, assay ≥ 99.8%, Pittsburgh, PA, USA, Cat. No. D119-1), ethanol (EtOH, 200 proof, ACS reagent, ≥99.5%, Sigma-Aldrich, St. Louis, MO, USA, Cat. No. 459844-1L), and hexanes (Certified ACS, Fisher Chemical, Pittsburgh, PA, USA, Cat. No. H292SK-4) were used as received without further purification. The commercial hexanes reagent is referred to as hexane throughout.

Nickel(II) nitrate hexahydrate (Ni(NO_3_)_2_·6H_2_O) and copper(II) nitrate trihydrate (Cu(NO_3_)_2_·3H_2_O) were purchased from Fisher Scientific (Waltham, MA, USA) and used as received without further purification.

**Figure 1:**
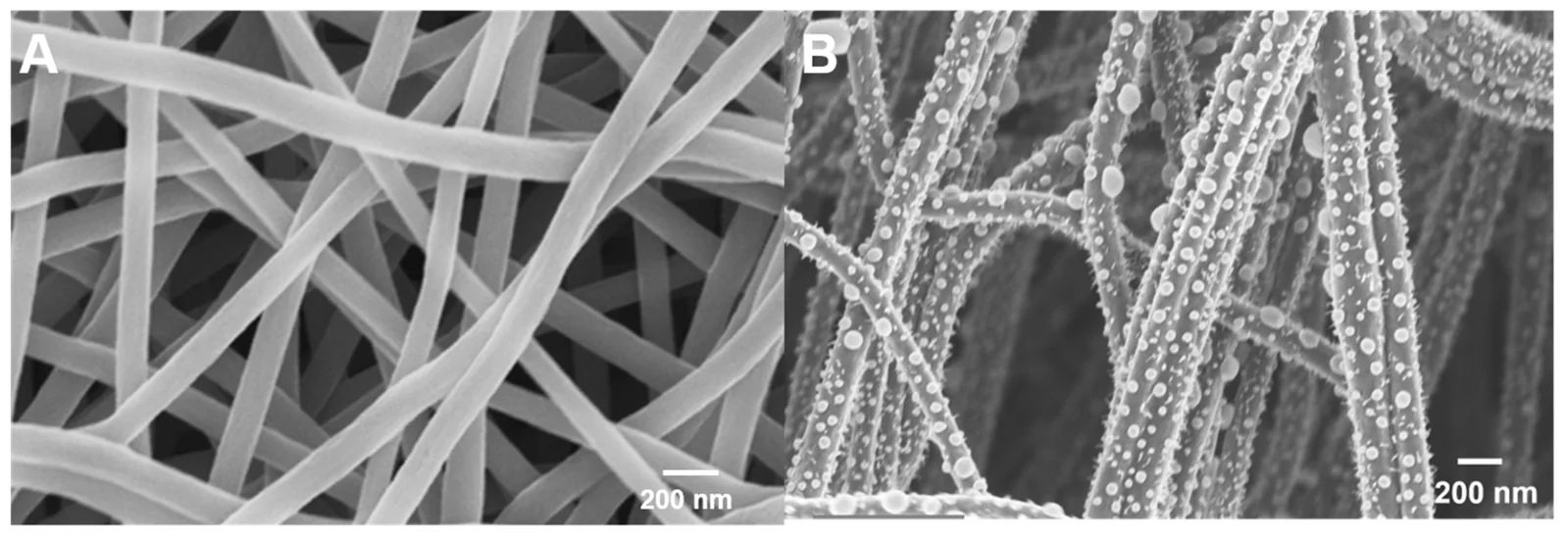
SEM images of (**A**) pristine CNFs and (**B**) Cu-containing nanoparticles prepared on CNFs from a 0.05 M ethanolic Cu(NO_3_)_2_ solution by single-solvent microwave CTS. Scale bars: 200 nm.

CNF preparation. An 8% wt. PAN solution was prepared by stirring 0.376 g of PAN homopolymer in 4.60 g of DMF overnight. The solution was electrospun vertically onto a grounded rotating drum collector at a flow rate of 0.5–1.0 mL·h^−1^, an applied voltage of approximately 14 kV, and a drum speed of approximately 150 rpm. The collected PAN mats were stabilized in flowing ambient air at 260 °C for 4 h, using a heating rate of 5 °C·min^−1^. The stabilized mats were then pyrolyzed under argon at approximately 20 sccm, heated to 800 °C at 5 °C·min^−1^, and held at 800 °C for 2 h. The resulting CNFs are shown in Figure 1A.

**Scheme 1:**
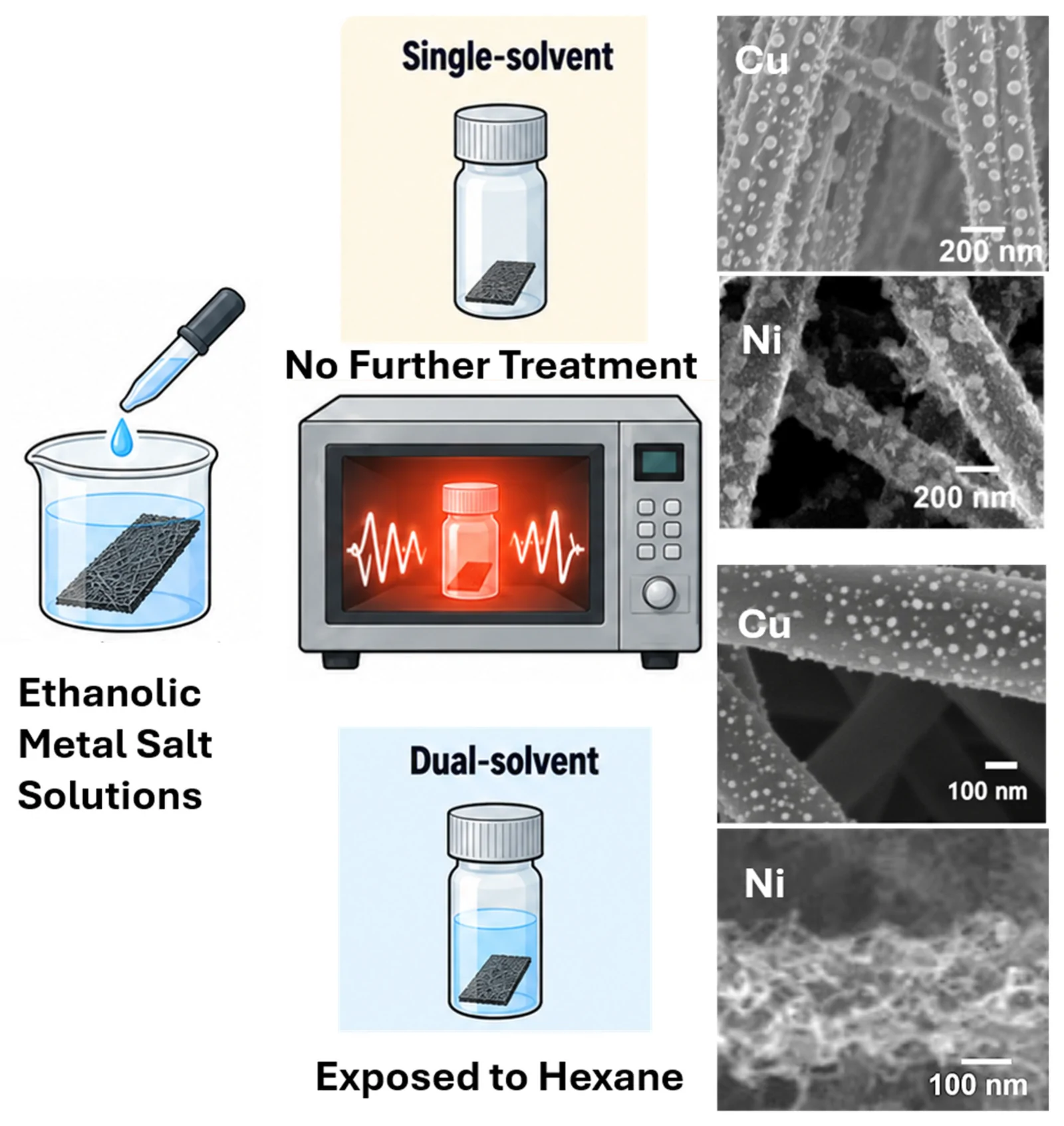
Schematic fabrication routes for producing metal nanostructures on carbon nanofibers by microwave CTS using single-solvent and dual-solvent methods.

Microwave CTS of CNFs. Copper(II) nitrate and nickel(II) nitrate precursor solutions were prepared separately in ethanol at concentrations of 0.01–0.20 M in increments of 0.01 M. CNF mats (1 cm × 3 cm) were immersed in the respective precursor solution for approximately 30 s to wet the porous fibrous network. This loading step involved contact with accessible fiber surfaces and the void space of the mat. For the single-solvent method, the loaded mat was transferred to a loosely capped glass vial without hexane. For the dual-solvent method, the loaded mat was transferred to a loosely capped glass vial containing 10–200 μL of hexane, varied in 10 μL increments. Precursor concentration and hexane volume were examined by holding one parameter constant while varying the other. The samples were irradiated in a conventional 2.45 GHz microwave oven for approximately 5 s. Following treatment, the samples were rinsed with ethanol to remove residual soluble salts and loosely bound residues and were air-dried overnight before characterization (Scheme 1).

Characterization. X-ray diffraction (XRD) patterns were recorded using a PANalytical Empyrean diffractometer (Malvern Panalytical, Almelo, The Netherlands) with Cu Kα radiation (λ = 1.5406 Å). X-ray photoelectron spectroscopy (XPS) measurements were performed using a Thermo Scientific ESCALAB 250Xi^+^ spectrometer (Thermo Fisher Scientific, Waltham, MA, USA). Scanning electron microscopy (SEM) images were acquired using a Zeiss Gemini ULTRA-55 field-emission scanning electron microscope (Carl Zeiss Microscopy GmbH, Oberkochen, Germany). Transmission electron microscopy (TEM), and scanning transmission electron microscopy-energy-dispersive X-ray spectroscopy (STEM-EDS) were performed using an FEI Tecnai F30 transmission electron microscope (Hillsboro, OR, USA). High resolution TEM images were obtained using a Thermo Scientific Talos F200i transmission electron microscope (Thermo Fisher Scientific, Waltham, MA, USA).

## 3. Results and Discussion

### 3.1. Effects of Dual-Solvent Processing on Cu-Containing Nanoparticles

To establish a morphological baseline for evaluating the effect of dual-solvent processing, the pristine CNF support and Cu-containing nanoparticles prepared by the single-solvent microwave CTS method were first characterized by SEM. Pristine CNFs had an average fiber diameter of 348 ± 97 nm (Figure 1A). Single-solvent microwave CTS of CNFs loaded with ethanolic copper nitrate produced quasi-spherical nanoparticles with an average diameter of 50 ± 20 nm and an average interparticle distance of 85 ± 20 nm (Figure 1B).

The particle diameter was comparable to the 56.8 ± 7.3 nm reported for Cu nanoparticles produced by Joule heating CTS [13]. This comparison provides a literature benchmark rather than a matched-process comparison. Although the 5 s microwave exposure used for this sample was longer than the 50–100 ms electrical pulses discussed for Joule heating CTS, extensive coarsening was not observed. A short heating interval may limit the time available for thermally activated surface diffusion, particle migration, and coalescence. Since the transient cooling rate was not measured, a quantitatively controlled quench cannot be established from these experiments.

Evaporation of ethanol in the single-solvent method can generate localized copper-salt-rich domains on the CNF surface, potentially limiting the number and spatial uniformity of nucleation sites. To promote a more homogeneous distribution of precursor nuclei and further restrict nanoparticle growth, an antisolvent-precipitation step was introduced to modify precursor distribution and limit subsequent aggregation. Antisolvent precipitation is an established bottom-up approach for nanoparticle synthesis [20,21]. Rapid contact between a solvent and an antisolvent sharply decreases precursor solubility, induces supersaturation, and promotes the formation of nanoscale precursor nuclei. In metal-based systems, this process can initially produce nanoscale metal-salt particles that are subsequently converted into metallic or metal-oxide nanoparticles through reduction or oxidation.

Based on this rationale, an appropriate antisolvent is needed to induce rapid precursor precipitation without disrupting the CNF architecture during microwave CTS. Several candidate secondary solvents were considered based on their compatibility with the CNF mat under microwave irradiation. Acetone, methanol, and acetonitrile caused substantial structural disruption during treatment, consistent with stronger microwave coupling, localized solvent heating, and rapid boiling. In contrast, nonpolar hexane couples weakly with microwave radiation, allowing energy deposition to remain concentrated primarily within the conductive carbon scaffold while preserving the fibrous architecture. Moreover, the low solubility of the metal-nitrate precursors in hexane provides the supersaturation required for antisolvent-induced precipitation. Hexane was therefore selected for the subsequent dual-solvent experiments.

**Figure 2:**
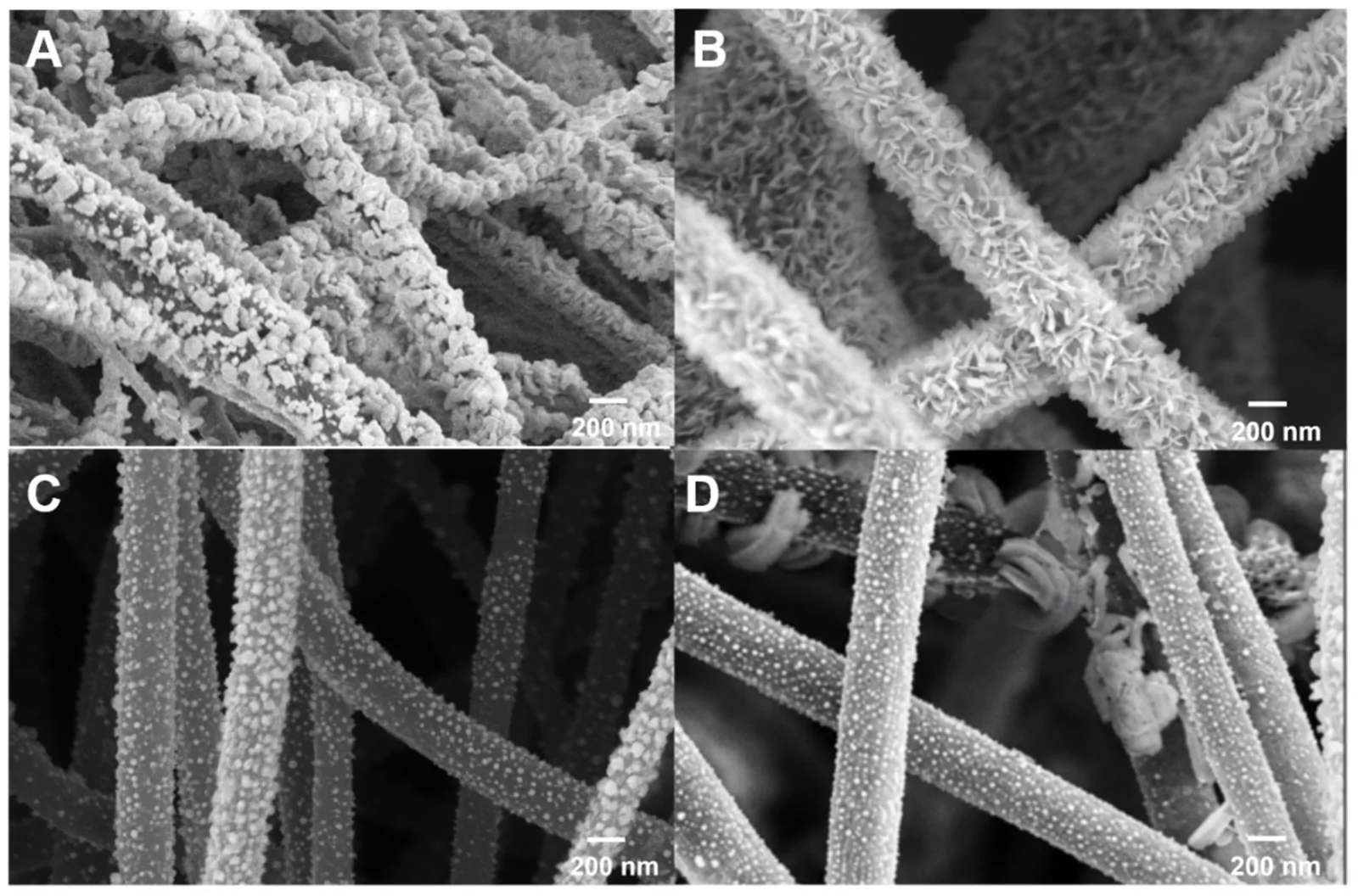
SEM images of Cu-containing nanostructures prepared by dual-solvent microwave CTS using 40 μL of hexane and Cu(NO_3_)_2_ concentrations of (**A**) 0.07 M, (**B**) 0.06 M, (**C**) 0.05 M, and (**D**) 0.04 M. Scale bars: 200 nm.

Using a fixed hexane volume of 40 μL, Cu-containing structures were prepared from Cu(NO_3_)_2_ concentrations of 0.04–0.07 M to examine the influence of precursor loading (Figure 2). At 0.07 M, strongly agglomerated features had a mean size of 44 ± 22 nm (Figure 2A), with edge-to-edge separations typically within 0–10 nm, indicating frequent contact between neighboring features. At 0.06 M, anisotropic flakes or needle-like structures formed, with an average length of 65 ± 25 nm and width of 18 ± 6 nm (Figure 2B). Their nearest-neighbor center-to-center spacing was 52 ± 15 nm, while edge-to-edge gaps commonly remained within 0–10 nm.

Further reducing the Cu(NO_3_)_2_ concentration to 0.05 M produced discrete, approximately spherical particles with a mean diameter of 33 ± 12 nm and a nearest-neighbor center spacing of 47 ± 14 nm (Figure 2C). These particles were smaller than those obtained under the single-solvent condition, which had an average diameter of approximately 50 ± 20 nm (Figure 1B). This reduction in particle size is consistent with antisolvent-induced precipitation producing more finely dispersed precursor domains, thereby increasing the number of nucleation sites and restricting subsequent particle growth. Changes in the local reaction environment and heat-transfer behavior may also suppress particle diffusion and coalescence during microwave processing. At 0.04 M, the smallest and most uniformly distributed particles were obtained, with a mean diameter of 30 ± 10 nm, a center-to-center spacing of 44 ± 9 nm, and an edge-to-edge gap of 14 ± 11 nm (Figure 2D). Thus, decreasing the precursor concentration progressively suppressed agglomeration and anisotropic growth.

**Figure 3:**
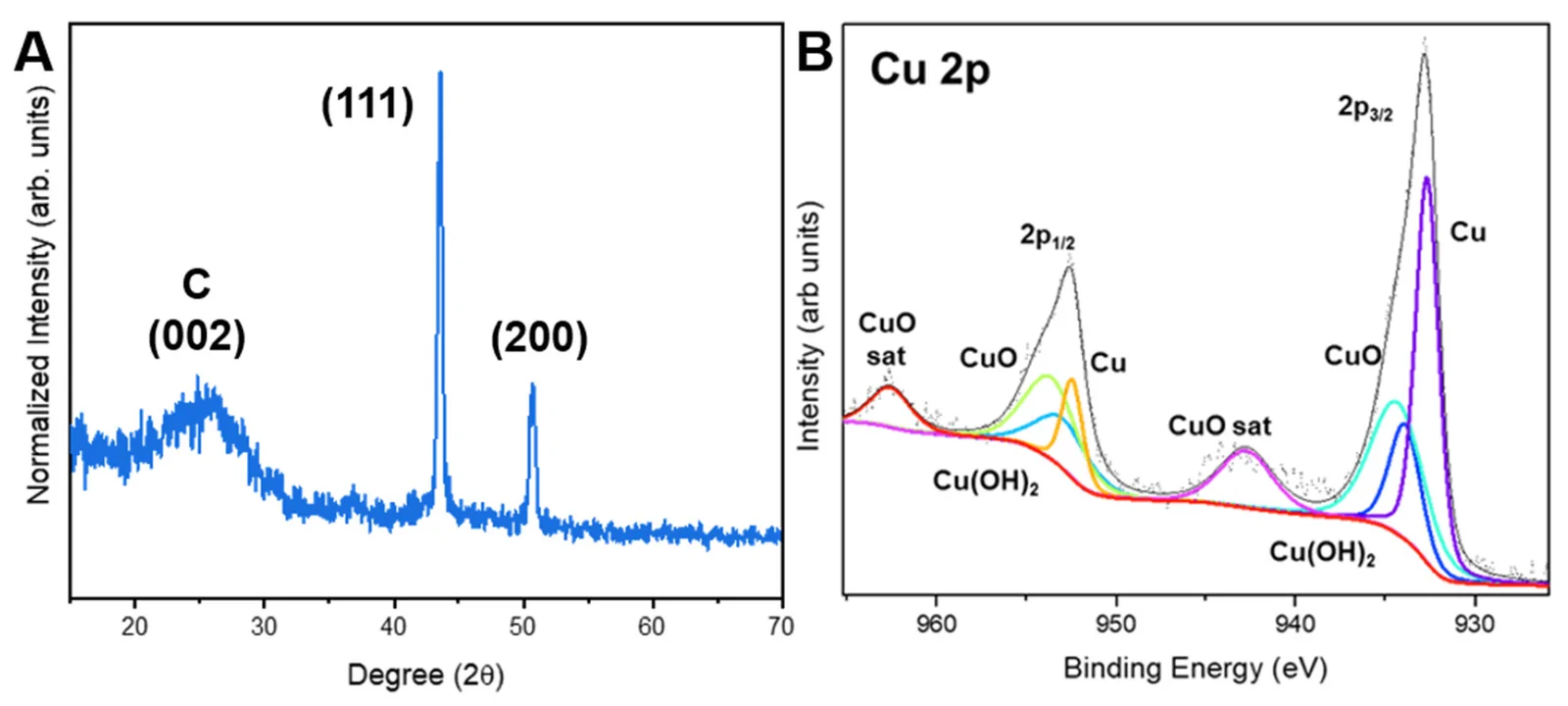
XRD and Cu 2p XPS characterization of Cu-containing CNFs prepared by dual-solvent microwave CTS. (**A**) XRD pattern with the broad CNF carbon reflection and fcc Cu(111) and Cu(200) reflections. (**B**) Cu 2p spectrum: gray/black points and line show the measured spectrum (black) and overall fit; purple and blue peaks represent the lower-binding-energy Cu contributions; cyan and light-green peaks repre-sent CuO-related components; orange peaks represent fitted Cu contributions in the 2p_1/2_ region; red curves represent Cu(OH)_2_-related contributions and the background; and the high-er-binding-energy red and purple features represent CuO shake-up satellites.

The Cu-containing nanoparticles prepared from a 0.05 M ethanolic Cu(NO_3_)_2_ solution using the dual-solvent method were characterized by XRD and XPS. The XRD pattern (Figure 3A) exhibits a broad reflection centered at approximately 26° 2θ, assigned to the C(002) plane of the CNF support. Sharp reflections at approximately 43.3° and 50.4° 2θ are indexed to the (111) and (200) planes of face-centered cubic metallic Cu, respectively, in agreement with the standard diffraction pattern for Cu (JCPDS/ICDD PDF No. 04-0836) and previously reported Cu nanoparticle diffraction data [16]. These reflections confirm the reduction in the Cu precursor and the formation of crystalline metallic Cu nanoparticles on the CNF surface.

The high-resolution Cu 2p XPS spectrum (Figure 3B) provides complementary surface information. The lower-binding-energy Cu 2p_3_/_2_ contribution at approximately 932–933 eV is consistent with Cu^0^ and/or Cu^+^, which cannot be distinguished reliably from this region alone. Higher-binding-energy contributions at approximately 933–935 eV and associated shake-up satellites indicate Cu^2+^-containing species, consistent with oxidized or hydroxylated surface contributions [21,22]. CuO-related surface signals demonstrate partial oxidation of the nanoparticles because the samples were characterized ex situ. Oxidation during cooling, handling, or storage is a plausible source of these signals; oxidation of Cu nanoparticles during air exposure has been reported [23]. Taken together, the XRD and XPS results demonstrate that the particles consist predominantly of crystalline metallic Cu with surface-oxidized Cu species, rather than phase-pure metallic Cu [16].

### 3.2. Ni-Catalyzed Carbon Nanotube Growth During Dual-Solvent Microwave Processing

**Figure 4:**
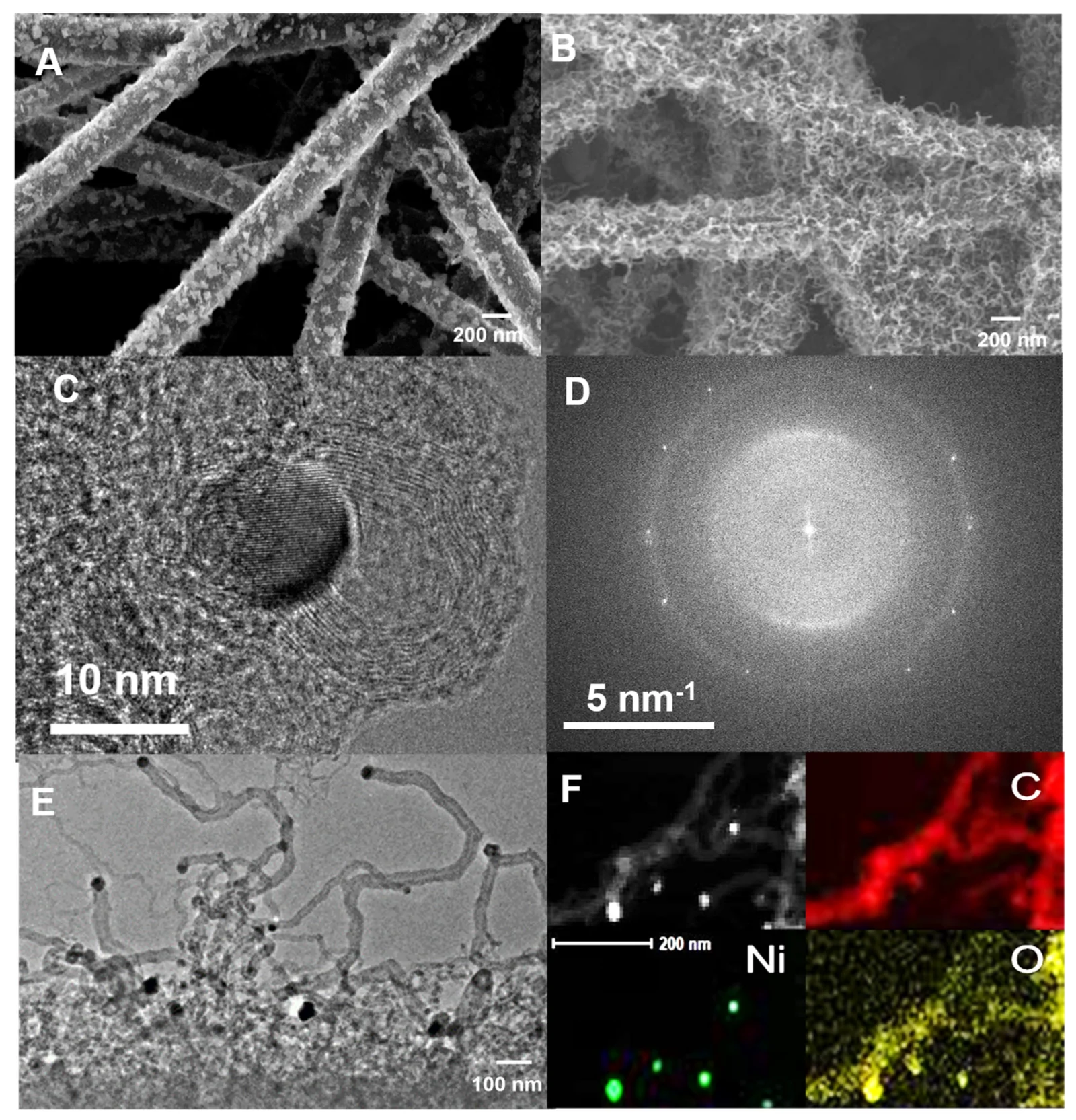
Microscopy of Ni-containing CNFs. (**A**) SEM image of aggregated particles produced by the single-solvent method. (**B**) SEM image of the filamentous network produced by the dual-solvent method. (**C**) HRTEM image and (**D**) corresponding FFT. (**E**) TEM image of curved carbon filaments associated with electron-dense particles. (**F**) STEM image and Ni, C, and O EDS maps showing the spatial association of Ni-containing particles with the carbonaceous structure. Scale bars are shown in each panel.

The influence of hexane on microwave CTS was further examined using CNFs treated with ethanolic Ni(NO_3_)_2_ solutions. Figure 4 compares the Ni-containing structures produced using single-solvent and dual-solvent processes. In the absence of hexane, the single-solvent process produced aggregated Ni-containing nanoparticles on the CNF surface, with an estimated mean diameter of 43 ± 16 nm (n = 50; Figure 4A). Larger fused aggregates of approximately 70–120 nm were also observed. In contrast, the addition of hexane produced a dense network of nanoscale filamentous structures with an apparent mean diameter of approximately 20 ± 7 nm (Figure 4B). Junctions and bundled regions were excluded from diameter analysis because they could not be reliably identified as individual filaments. Subsequent TEM analysis confirmed that these filamentous structures were CNTs.

The distinct morphologies observed for Ni- and Cu-containing CNFs can be attributed to differences in their catalytic interactions with carbon during microwave CTS. Ni is an established catalyst for CNT growth because of its ability to activate hydrocarbon molecules, accommodate carbon at elevated temperatures, and facilitate carbon transport and graphitic structure formation [24,25]. Reactive carbon species generated during microwave CTS may diffuse along the Ni particle surface or through subsurface regions before being incorporated into growing CNT walls (Figure 4C). In contrast, Cu exhibits substantially lower carbon solubility and generally weaker catalytic activity for hydrocarbon decomposition, making sustained carbon incorporation and CNT nucleation less favorable under the short processing conditions employed here [26,27]. The formation of CNTs only in the Ni-containing dual-solvent samples suggests that hexane may also provide reactive carbon species through thermal or Ni-catalyzed decomposition, in addition to its function as an antisolvent. However, the carbon source and growth pathway remain to be confirmed experimentally. Although Cu has been reported to catalyze CNT growth under appropriately controlled conditions [27], the present results indicate that Ni provides a more favorable combination of carbon activation, transport, and graphitic nucleation during rapid microwave CTS.

The HRTEM image in Figure 4C shows a crystalline nanoparticle adjacent to short, curved graphitic layers, consistent with localized carbon ordering associated with the Ni-containing structures. The heterogeneous layer orientations are consistent with partially ordered or turbostratic graphitic carbon. The corresponding fast Fourier transform (FFT; Figure 4D) contains features at approximately 4.8–5.0 nm^−1^. Using d = 1/g, where g is spatial frequency, these features correspond to real-space spacings of approximately 0.20–0.21 nm, consistent with fcc Ni(111), for which the expected spacing is approximately 0.203 nm. Because other Ni-containing phases can produce similar spacings, the assignment is considered together with XRD rather than established from this FFT alone [28]. A broader diffuse feature near 3 nm^−1^ corresponds to approximately 0.33–0.34 nm, consistent with the (002) interlayer periodicity of poorly ordered graphitic carbon.

The TEM image in Figure 4E shows curved CNTs associated with electron-dense nanoparticles. Some particles are located near CNT termini, consistent with a possible tip-growth mechanism; however, their distribution does not establish a single growth mode. STEM-EDS mapping (Figure 4F) supports the spatial association of Ni-containing particles with carbonaceous structures. Oxygen detected in the analyzed region may originate from surface oxidation of Ni-containing particles or oxygen-containing species on the support. EDS alone does not determine the chemical state or specific origin of this oxygen.

**Figure 5:**
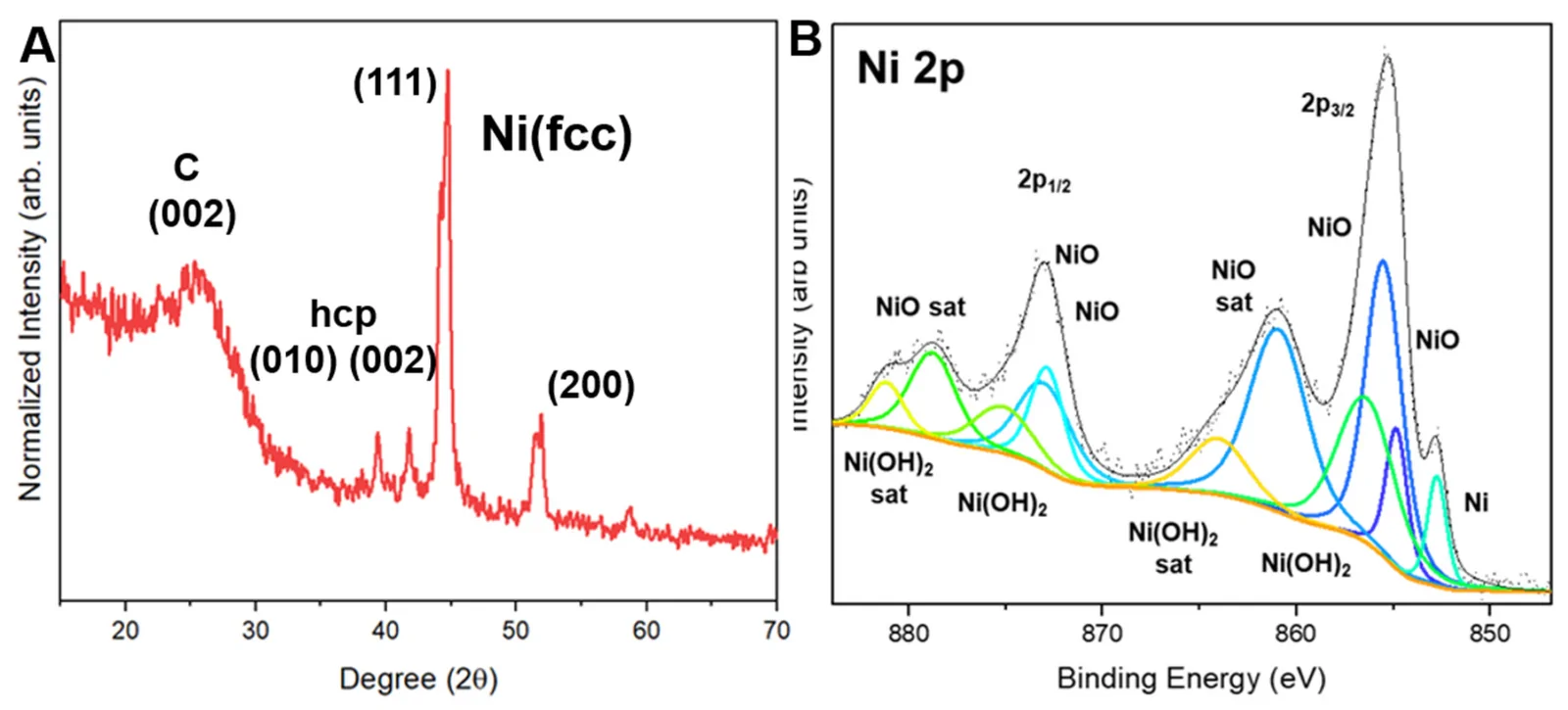
XRD and Ni 2p XPS characterization of Ni-containing CNFs prepared by dual-solvent microwave CTS. (**A**) XRD pattern with fcc Ni reflections and additional reflections assigned tentatively to an hcp-like Ni-containing phase. The plotted hcp labels are not a definitive distinction between hcp Ni and Ni_3_C. (**B**) Ni 2p spectrum: gray/black points and line show the measured spectrum and overall fit; green and violet peaks include low-binding-energy reduced-Ni contributions; blue and cyan peaks represent NiO-associated Ni^2+^ components; green/cyan components at higher binding energy and yellow components depict additional fitted Ni^2+^ features and shake-up satellites, including NiO and Ni(OH)_2_-related satellites; orange curves show Ni(OH)_2_-related contributions. Individual component colors distinguish overlapping fitted features, rather than inde-pendently establishing distinct chemical phases.

The XRD pattern of the dual-solvent Ni-containing sample shows reflections at 44.7° and 51.6° 2θ, assigned to fcc Ni(111) and Ni(200), respectively (Figure 5A). Additional reflections at 39.4°, 41.8°, 44.2°, and 58.8° are compatible with a hexagonal Ni-containing phase. Metastable hcp Ni has been reported under solvent-assisted or rapid-quenching conditions [29,30,31]. However, overlapping hcp Ni and Ni_3_C reflections limit discrimination by XRD alone, particularly in carbon-rich samples [28]. The feature near 44.2° also overlaps the neighboring fcc Ni(111) reflection. The additional reflections are therefore assigned conservatively to an hcp-like phase that may comprise metastable hcp Ni, Ni_3_C, or both.

The Ni 2p XPS spectrum (Figure 5B) contains a low-binding-energy Ni 2p_3_/_2_ component at 852.73 eV, consistent with metallic Ni, together with broader Ni^2+^-associated components and shake-up satellites [32]. These features are consistent with NiO and/or Ni(OH)_2_ at the surface, although their relative contributions cannot be established from individual peak positions alone. Oxidation or hydroxylation during post-synthesis handling is a possible explanation, but oxidation during processing is not excluded. Together, XRD and XPS support crystalline fcc Ni, a possible hcp-Ni/Ni_3_C contribution, and oxidized surface species.

Taken together, microscopy, diffraction, and surface analysis distinguish two outcomes of dual-solvent microwave CTS: supported Cu-containing particles with concentration-dependent morphology, and Ni-containing particles associated with a CNT network. The contrast supports a role for metal identity in nanostructure evolution. Antisolvent-assisted precursor redistribution and metal-dependent carbon chemistry provide a plausible interpretation, while the local thermal history, carbon source, and growth pathway remain to be established directly.

Although the dual-solvent microwave CTS approach provides a rapid route to supported metal nanoparticles and CNT-containing structures, several aspects require further investigation. Future studies should quantify the transient temperature and cooling rates during microwave treatment, clarify the role of hexane through appropriate carbon-source control experiments, and examine the scalability and reproducibility of the process under more precisely controlled atmospheres. Extending the method to additional mono- and multimetallic systems, together with application-specific testing in catalysis, sensing, or electrochemical platforms, will help determine the broader utility of this processing strategy.

## 4. Conclusions

This study demonstrates that the dual-solvent microwave CTS method provides a rapid and effective approach for fabricating metal-containing nanostructures on CNFs. The addition of hexane, an anti-solvent of metal salts, improved the dispersion of Cu-containing nanoparticles, reducing their average diameter from 50 ± 20 nm to 33 ± 12 nm at 0.05 M precursor concentration. In contrast, Ni-containing nanoparticles promoted the formation of CNTs with an average diameter of 20 ± 7 nm. This difference is attributed to the higher catalytic activity of Ni toward hydrocarbon decomposition, its greater carbon solubility, and its ability to facilitate carbon transport and graphitic structure formation. Hexane may additionally supply reactive carbon species during CNT growth, although its contribution requires further verification. These findings demonstrate that metal identity and solvent conditions strongly influence nanostructure evolution during microwave CTS, offering a versatile strategy for engineering metal–carbon nanocomposites with tunable morphologies for potential catalytic and electrochemical applications.

## Data Availability

The original contributions presented in this study are included in the article. Further inquiries can be directed to the corresponding author.

## Abbreviations

The following abbreviations are used in this manuscript:

PAN	Polyacrylonitrile
CNF	Carbon nanofiber
CNT	Carbon nanotube
CTS	Carbothermal shock
DMF	N,N-Dimethylformamide
EDS	Energy-dispersive X-ray spectroscopy
fcc	Face-centered cubic
hcp	Hexagonal close-packed
SEM	Scanning electron microscopy
TEM	Transmission electron microscopy
STEM	Scanning transmission electron microscopy
XPS	X-ray photoelectron spectroscopy
XRD	X-ray diffraction
